# Supplementary material for: Ultrafast photoluminescence and multiscale light amplification in nanoplasmonic cavity glass
Source: Nat Commun. 2024 Apr 17;15:3309. doi: 10.1038/s41467-024-47539-3 (PMC11024168; doi:10.1038/s41467-024-47539-3)
Supplement: Supplementary file 1 — Supplementary Information [file 41467_2024_47539_MOESM1_ESM.pdf]

Supplementary Material

Ultrafast Photoluminescence and Multiscale Light Amplification in Nanoplasmonic Cavity Glass

Piotr Piotrowski[1,2*], Marta Buza[3], Rafał Nowaczyński[2,4], Nuttawut Kongsuwan[5,6], Hańcza B. Surma[1,3], Paweł Osewski[3], Marcin Gajc[3], Adam Strzep[7], Witold Ryba-Romanowski[7], Ortwin Hess*[8], Dorota A. Pawlak*[1,2,3]

[1]Centre of Excellence ENSEMBLE[3] sp. z o.o., Wolczynska 133, 01-919 Warsaw, Poland
[2]Faculty of Chemistry, University of Warsaw, Pasteura 1, 02-093 Warsaw, Poland
[3]Łukasiewicz Research Network - Institute of Microelectronics and Photonics, Wolczynska 133, 01-919 Warsaw, Poland
[4]Faculty of Materials Science and Engineering, Warsaw University of Technology, Woloska 141, 02-507 Warsaw, Poland
[5]Quantum Technology Foundation (Thailand), 98 Soi Ari, Bangkok 10110, Thailand
[6]Thailand Center of Excellence in Physics, Ministry of Higher Education, Science, Research and Innovation, Bangkok 10400, Thailand
[7]Institute of Low Temperature and Structure Research PAS, Okolna 2, 50-422 Wroclaw, Poland
[8]School of Physics and CRANN Institute, Trinity College Dublin, Dublin 2, Ireland

**corresponding authors:* *piotr.piotrowski@ensemble3.eu*
dorota.anna.pawlak@ensemble3.eu
ortwin.hess@tcd.ie

This paper is dedicated to the memory of our dear co-worker and friend Hańcza Barbara Surma, who passed away while this paper was being completed.

Supplementary Note 1 – Absorbance of the glass composites

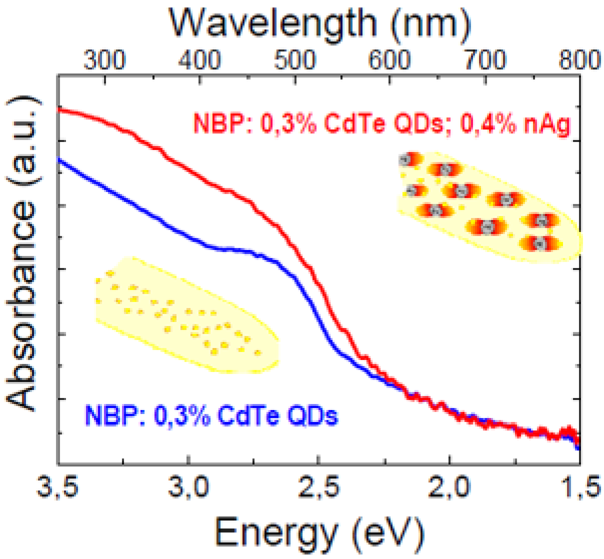

Supplementary Figure 1 | Absorbance spectrum of the glass nanocomposites: NBP:CdTe(0.3) (blue) and NBP:CdTe(0.3)nAg(0.4) (red).

Supplementary Note 2 – Characterization of CdTe quantum dots

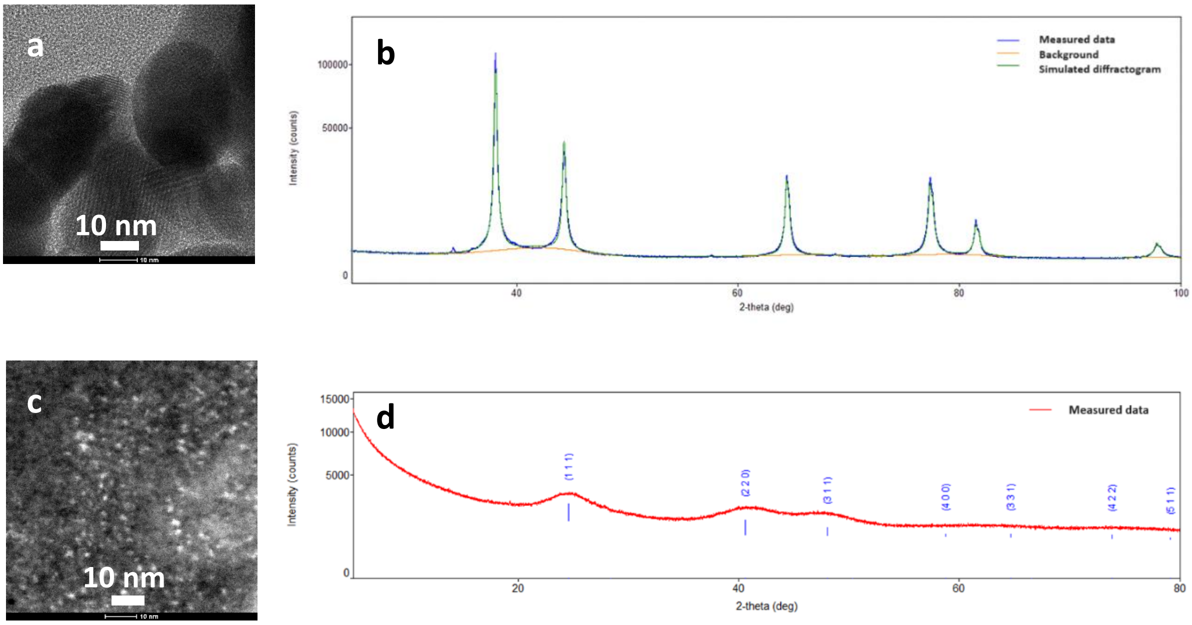

Supplementary Figure 2 | a) TEM image and **b)** powder X-ray diffractogram for nAg; **c)** TEM image and **d)** powder X-ray diffractogram for CdTe QDs.

Supplementary Note 3 - Finite-Difference Time-Domain Simulations

Fluorescence emission and optical decay of CdTe QDs inside the nanocomposites were simulated by performing 3D full-wave FDTD simulations using commercial software, Lumerical FDTD version 8.18.1298. The NBP glass matrix was modelled as a background dielectric material with refractive index 1.517 whereas AgNPs were modelled as spheres with diameters of 20 nm. The permittivity of silver was taken from experimental data by Johnson and Christy [1]. The size of the simulated volume was 1 μm × 1 μm × 1 μm. The conformal meshing scheme was used between dielectric interfaces, but not on metal interfaces, with a maximum step size of 10 nm in all directions. Small mesh refinements of 2 nm and 0.2 nm were used around the AgNPs and CdTe QDs, respectively.

Fluorescence emission of an emitter was modelled by two separate simulations where one simulated excitation on the emitter and the other simulated emission from the emitter. In excitation simulations, AgNPs were excited by a plane wave, and the excitation rate was obtained by the square of the enhanced electric field $\mathbf{E}$, i.e. $\gamma_{\mathrm{exc}} \propto |\mathbf{E}|^2$. In emission simulations, the emitters were modelled as simple electric dipole emitters. The total decay rate γ_{tot} was calculated by the total field power emitted by the emitter whereas the radiative decay rate γ_{rad} was calculated by the field power that radiates to the far-field.

Simulations of Enhanced Fluorescence Emission

When an emitter is placed in close proximity to a single AgNP, the emitter's optical properties are altered due to its interaction with the plasmonic field from the nanoparticle. This interaction enhances the radiative decay rate of the emitter γ_{rad} and also dissipates energy via Ohmic loss γ_{nr} [2]. For an emitter with a vacuum radiative decay of γ_0, Supplementary Figure 3 shows the radiative decay enhancement $\tilde{\gamma}_{\mathrm{rad}} = \gamma_{\mathrm{rad}}/\gamma_0$ and the total decay enhancement $\tilde{\gamma}_{\mathrm{tot}} = \gamma_{\mathrm{tot}}/\gamma_0 = (\gamma_{\mathrm{rad}} + \gamma_{\mathrm{nr}})/\gamma_0$ of a quantum emitter placed at distance z = 2, 4, 10 nm from a 20-nm AgNP. The results reveal that the emitter interacts more strongly with the plasmonic field when its dipole moment is aligned toward the nanoparticle, as shown in Supplementary Figures 3a and 3b. The radiative decay $\tilde{\gamma}_{\mathrm{rad}}$ can be strongly enhanced near 410 nm, which corresponds to the nanoparticle's first- order plasmonic resonance [3]. On the other hand, the emitter will be rapidly quenched if its transition wavelength lies below 400 nm due to the collective higher plasmonic modes which are known as a pseudomode of the nanoparticle [4].

During the process of fabricating a nanocomposite, AgNPs and CdTe QDs are randomly distributed inside the glass matrix. Due to the random nature of the process, some CdTe QDs

could, by chance, be positioned in a nanoscale gap (nanocavity) between two AgNPs. At such a nanocavity, plasmonic modes of the two AgNPs hybridize and form dimer plasmonic modes that are known to provide extreme field confinement and enhancement [5]. Supplementary Figure 4 shows how a nanocavity modifies the decay rates of an emitter when it is placed at the nanocavity's centre. As shown in Supplementary Figure 4a, a nanocavity with gap size of $2z = 4$ nm provides a 20-fold increase in radiative decay for the emitter with its dipole moment aligned parallel to the gap, compared to the single AgNP case in Supplementary Figure 3a. On the other hand, the emitter's emission is suppressed if its dipole moment is aligned perpendicular to the gap, as shown in Supplementary Figure 4c,d. In addition, the first-order hybridized plasmonic mode is also spectrally located at higher wavelength (445 nm) than that of the single AgNP (410 nm). Since CdTe QDs' emission wavelength is at 513 nm, CdTe QDs are much closer to resonance with the first-order dimer plasmonic mode and experience a significant larger field enhancement.

To illustrate how the emitter's optical properties vary with distance z, Supplementary Figures 5 and 6 show 2D spectral plots of radiative decay enhancement $\tilde{\gamma}_{\text{rad}}$, total decay enhancement $\tilde{\gamma}_{\text{tot}}$ and excitation enhancement $\tilde{\gamma}_{\text{exc}}$ of the emitter placed near a AgNP and inside Ag dimer nanocavity, respectively. Note that $\tilde{\gamma}_{\text{exc}}$ in Supplementary Figure 6f is zero as the symmetry of the arrangement of two AgNPs cancels the excitation field on the emitter.

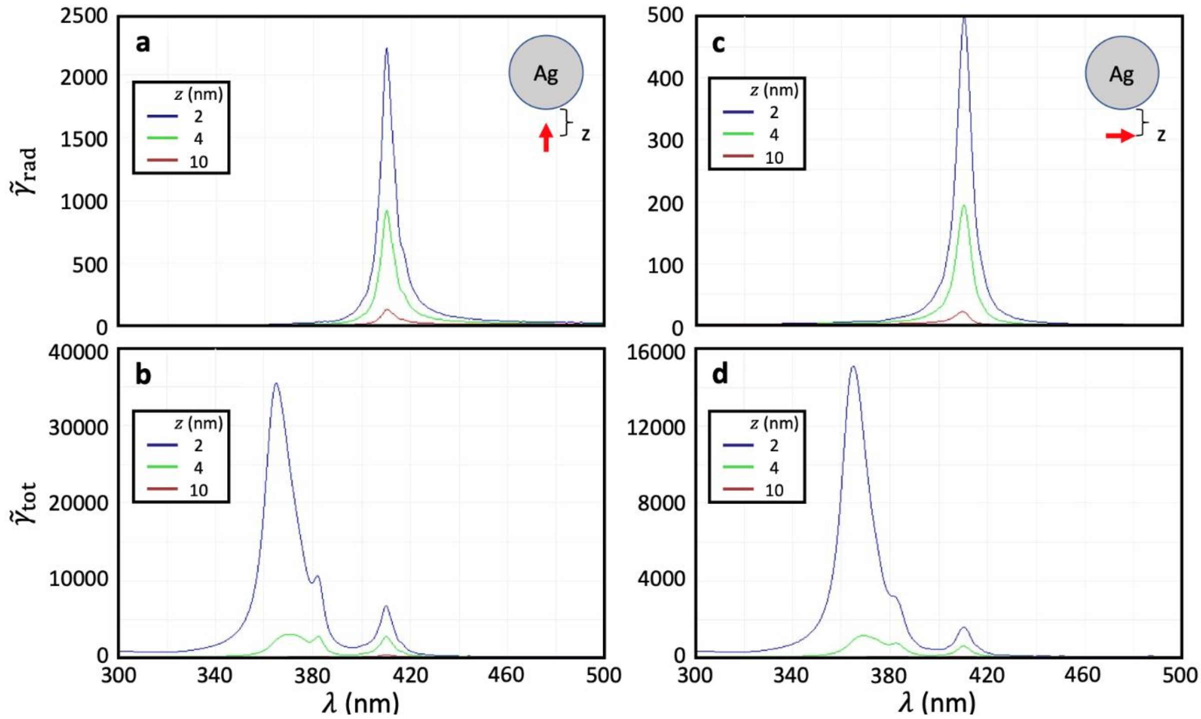

Supplementary Figure 3 | a,c) Radiative decay enhancement $\tilde{\gamma}_{\text{rad}}$ and **b,d)** total decay enhancement (Purcell factor) $\tilde{\gamma}_{\text{tot}}$ of a quantum emitter placed at distance $z = 2, 4, 10$ nm from a 20-nm AgNP. The dipole moment of the emitter is aligned **a,b)** parallel to the z-axis and **c,d)** perpendicular to the z-axis.

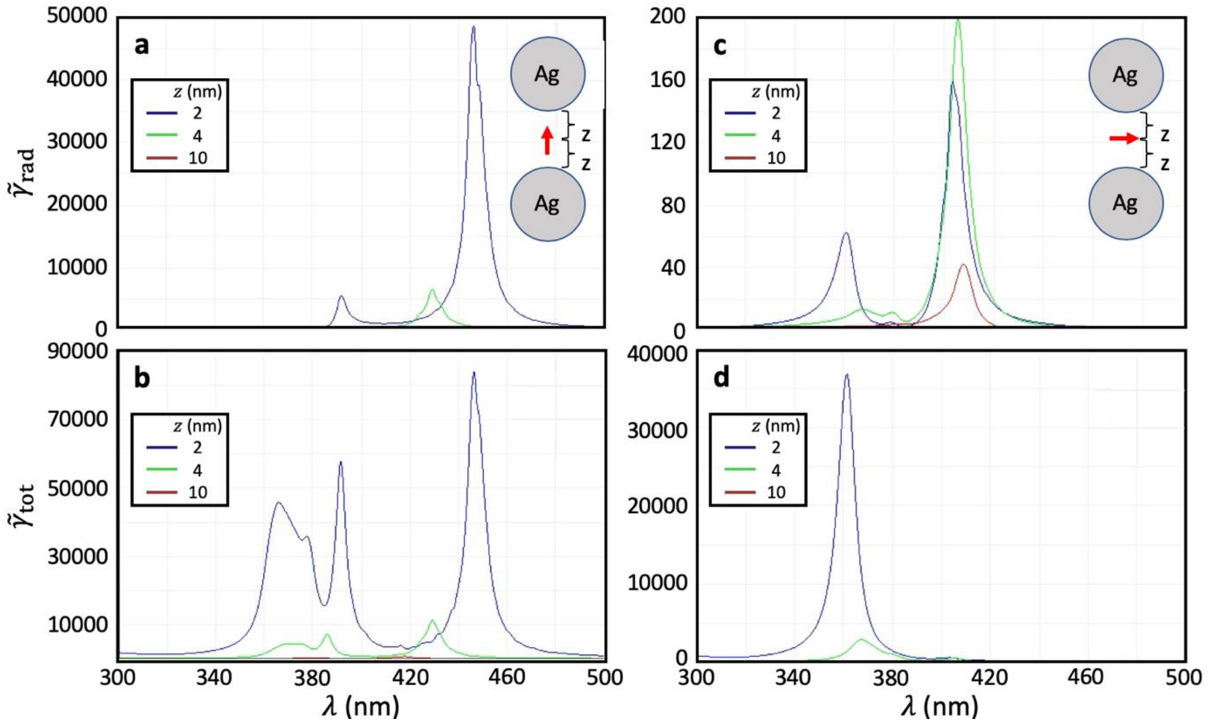

Supplementary Figure 4 | a,c) Radiative decay enhancement $\tilde{\gamma}_{\mathrm{rad}}$ and **b,d)** total decay enhancement (Purcell factor) $\tilde{\gamma}_{\mathrm{tot}}$ of a quantum emitter placed at the middle of a Ag dimer nanocavity with diameter 20 nm and gap size $2z$ where $z = 2$, 4, 10 nm. The dipole moment of the emitter is aligned **a,b)** parallel to the gap and **c,d)** perpendicular to the gap.

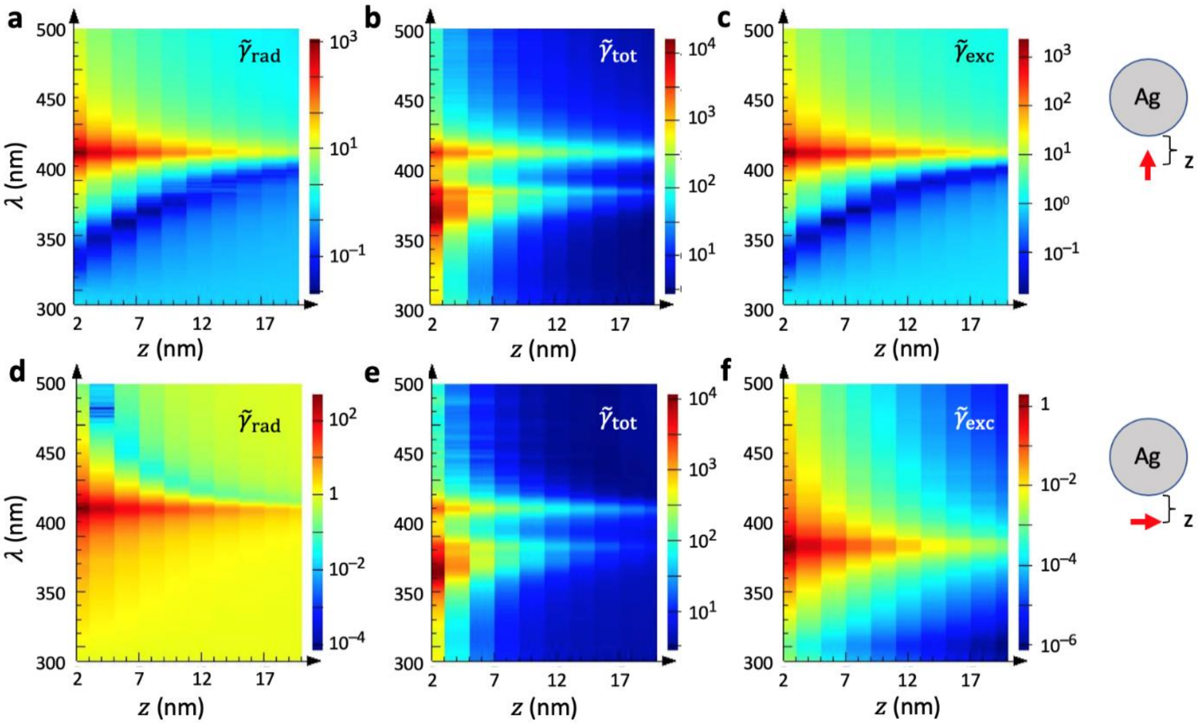

Supplementary Figure 5 | a,d) Radiative decay enhancement $\tilde{\gamma}_{\mathrm{rad}}$, **b,e)** total decay enhancement (Purcell factor) $\tilde{\gamma}_{\mathrm{tot}}$ and **c,f)** excitation enhancement $\tilde{\gamma}_{\mathrm{exc}}$ of a quantum emitter placed at distance z from a 20-nm AgNP. The dipole moment of the emitter is aligned **a,b,c)** parallel to the z-axis and **d,e,f)** perpendicular to the z-axis.

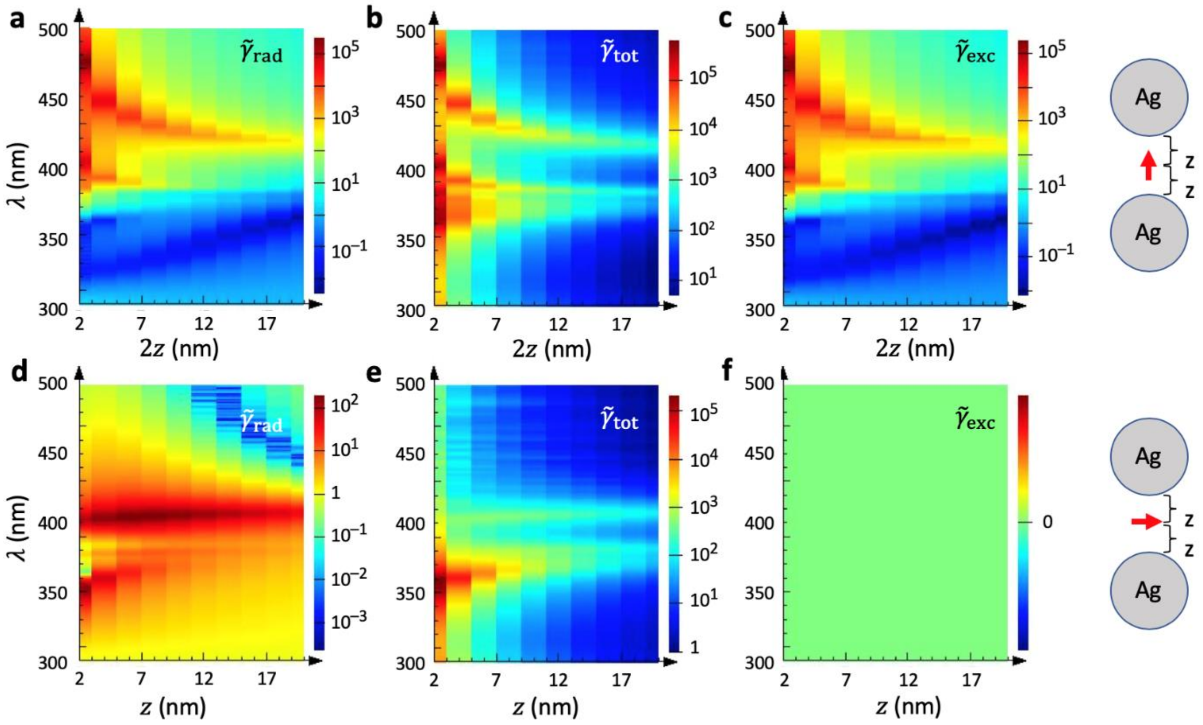

Supplementary Figure 6 | a,d) Radiative decay enhancement $\tilde{\gamma}_{\mathrm{rad}}$, **b,e)** total decay enhancement (Purcell factor) $\tilde{\gamma}_{\mathrm{tot}}$ and **c,f)** excitation enhancement $\tilde{\gamma}_{\mathrm{exc}}$ of a quantum emitter placed at the middle of a Ag dimer nanocavity with diameter 20 nm and gap size $2z$. The dipole moment of the emitter is aligned **a,b,c)** parallel to the gap and **d,e,f)** perpendicular to the gap.

References:

[1] P.B. Johnson, R.W. Christy, Optical constants of the noble metals, Phys. Rev. B. 6 (1972) 4370–4379. doi:10.1103/PhysRevB.6.4370.

[2] P. Anger, P. Bharadwaj, L. Novotny, Enhancement and quenching of single-molecule fluorescence, Phys. Rev. Lett. 96 (2006) 113002. doi:10.1103/PhysRevLett.96.113002.

[3] N. Kongsuwan, A. Demetriadou, R. Chikkaraddy, F. Benz, V.A. Turek, U.F. Keyser, J.J. Baumberg, O. Hess, Suppressed Quenching and Strong-Coupling of Purcell-Enhanced Single-Molecule Emission in Plasmonic Nanocavities, ACS Photonics. 5 (2018) 186–191. doi:10.1021/acsphotonics.7b00668.

[4] A. Delga, J. Feist, J. Bravo-Abad, F.J. Garcia-Vidal, Quantum emitters near a metal nanoparticle: Strong coupling and quenching, Phys. Rev. Lett. 112 (2014) 253601. doi:10.1103/PhysRevLett.112.253601.

[5] R. Chikkaraddy, B. De Nijs, F. Benz, S.J. Barrow, O.A. Scherman, E. Rosta, A. Demetriadou, P. Fox, O. Hess, J.J. Baumberg, Single-molecule strong coupling at room temperature in plasmonic nanocavities, Nature. 535 (2016) 127–130. doi:10.1038/nature17974.
